# The prevalence of panic disorder in chronic obstructive pulmonary disease: a systematic review, meta-analysis, and meta-regression

**DOI:** 10.1186/s13643-026-03102-3

**Published:** 2026-02-16

**Authors:** Bronwyn Todd, Guillaume Foldes-Busque, Kimberley L. Way, Khang Duy Ricky Le, Marlies Alvarenga, Christopher M. Celano, Jeff C. Huffman, Phillip J. Tully

**Affiliations:** 1 School of Psychology, La Trobe University, Flora Hill, Bendigo, VIC Australia; 2 School of Psychology, Université Laval Québec, Quebec, Canada; 3 Quebec Heart and Lung Institute, Québec, Canada; 4 Institute for Physical Activity and Nutrition, School of Exercise and Nutrition Sciences, Deakin University, Geelong, VIC Australia; 5 Exercise Physiology and Cardiovascular Health Lab, Division of Cardiac Prevention and Rehabilitation, University of Ottawa Heart Institute, Ottawa, ON Canada; 6 School of Medicine, Faculty of Health, Deakin University, Geelong, Victoria Australia; 7 Department of General Surgical Specialties, The Royal Melbourne Hospital, Melbourne, Victoria Australia; 8 Institute of Health and Wellbeing, Federation University Australia, Berwick, Victoria Australia; 9 Victorian Heart Institute, Melbourne, Victoria Australia; 10 Department of Psychiatry, Harvard Medical School, Boston, MA USA; 11 Department of Psychiatry, Massachusetts General Hospital, Boston, MA USA; 12 School of Psychology, Deakin University, Geelong, Victoria 3216 Australia; 13 School of Medicine, Adelaide University, Adelaide, SA Australia

**Keywords:** Chronic obstructive pulmonary disease, Chronic obstructive airway disease, Panic disorder, Panic attack, Meta-analysis

## Abstract

**Background:**

Chronic obstructive pulmonary disease (COPD) symptoms of dyspnea and chest tightness overlap with some symptoms of panic attacks, the hallmark feature of panic disorder (PD). Our objective was to quantify PD prevalence in COPD from a systematic review and meta-analysis.

**Methods:**

A database search from inception to January 2025 was performed using five electronic databases. Eligible studies utilized structured clinical psychiatric interviews to identify PD in adult populations with COPD derived from inpatient, outpatient, or general population sampling.

**Results:**

Twenty-one studies met inclusion criteria, with most from Asia (*k* = 9), reporting data from 1847 persons with COPD, 860 healthy controls, and 450 persons with comorbidities other than COPD. The prevalence of PD in persons with COPD was 12.5% (95% confidence interval [CI] 8.2–18.7, *I*^2^ = 90%), revised to 8.1% (95% *CI* 5.7–11.6, *I*^2^ = 73%) after the exclusion of *k* = 5 outliers. In case-control studies, PD was more prevalent in COPD patients (*k* = 9, 9.7%; 95% CI 5.9–15.4, *I*^2^ = 69%) than healthy controls (*k* = 6, 2.8%; 95% *CI* 1.7–4.6, *I*^2^ = 0%). There was no evidence to suggest a higher PD prevalence than in other medical conditions (*k* = 5, 4.8%; 95% *CI* 1.8–12.0, *I*^2^ = 47%).

**Conclusions:**

The pooled estimates of PD in COPD were higher than the general population but markedly lower than suggested by prior narrative reviews. Further research needs to elucidate whether the characteristic symptoms of panic in COPD are similar to PD in non-COPD populations and, secondly, whether they lead to differential healthcare resource utilization and portend a higher risk for adverse outcomes.

**Systematic review registration:**

CRD42024559743.

**Supplementary Information:**

The online version contains supplementary material available at 10.1186/s13643-026-03102-3.

## Background

Chronic obstructive pulmonary disease (COPD) is an irreversible obstructive lung condition characterized by respiratory symptoms that may include dyspnea, wheezing, chest tightness, cough, and sputum production [1]. COPD remains a leading cause of death and disability globally [2], with many patients experiencing exacerbations that necessitate inpatient hospital care [3]. Exacerbations of COPD can be perceived as frightening, with patients recalling intense fear [4, 5] and sensations akin to suffocation or choking to death [6]. This supports a cognitive component to dyspnea, comprising perceptions of discomfort, unpleasantness, and negative emotional impact [7]. Qualitative research indicates that the management of anxiety, panic, and breathlessness is among COPD patients’ leading priorities in cardiopulmonary rehabilitation [8]. The cardiopulmonary symptoms of dyspnea and chest tightness in COPD closely overlap those experienced during panic attacks, the hallmark feature of panic disorder (PD, [9]). Research showing COPD symptom relief immediately upon the arrival of paramedics prior to medical intervention [5] suggests that accurate diagnosis and management of PD in COPD could help reduce the health system burden attributable to PD [10, 11].

Among patients with concurrent cardiopulmonary disease, breathlessness may occur during exercise or moderate exertion, leading to a panic attack [5, 6, 12]. Explanations put forward to explain the high preponderance to PD in COPD include heightened sensitivity to unpleasant physical sensations and symptoms such as dyspnea [13, 14] and catastrophic cognitions in the face of real threat such as an exacerbation of COPD [6]. However, the prevalence of PD in COPD patients remains poorly characterized. The extant literature includes self-report measures of anxiety symptoms where the terms “anxiety disorder,” “panic symptoms,” “panic attack,” and “panic disorder” are used interchangeably and seldom correctly, mislabeling nonspecific anxiety symptoms as panic symptoms or PD (for reviews, see either [15, 16]). Indeed, while patients with an anxiety disorder will often experience nonspecific anxiety symptoms of varied intensity, a diagnosis of PD refers to the presence of specific symptoms, including the occurrence of panic attacks, which persist over time and cause clinically significant distress or impairment in important areas of functioning. Relatedly, a panic attack itself is not a standalone diagnosable condition [9], even though its presence as recurrent unexpected attacks is essential for a PD diagnosis.

In COPD, a previous literature review reported PD with or without agoraphobia prevalence as ranging from 0 to 41% [17]; however, a meta-analysis was not performed. Another review mentioned prevalence rates as high as 67% [15]; however, this figure was based on only nine COPD patients and a convenience sample where not all respiratory referral cases underwent psychiatric interview [18], raising the possibility that the prevalence of PD is overestimated. Narrative reviews have promulgated considerably high PD prevalence estimates up to 50% [15–17, 19–21]. It is also suggested that COPD inpatients have higher PD prevalence than other medical conditions [22]; however, no prior review has quantified this with meta-analysis. Extant reports as high as 67% PD prevalence in COPD appear concerning. Accurate estimates of global prevalence and comorbidity are helpful for healthcare resource planning, clinical interventions and policy development [23]. Crucially, diagnostic rules are explicit that a PD diagnosis should not be made when the disturbance is clearly attributable to the physiological effects of a medical condition [9]. Notably, cardiopulmonary disorders are one of only two exemplar conditions mentioned in the DSM-5’s diagnostic criteria for PD [9], the other being hyperthyroidism. Collectively, this suggests that not only are PD prevalence reports in COPD grossly overestimated but they may also be conceptually spurious.

Taking into consideration past limitations, the objective of this study was to perform a systematic review and meta-analysis of PD prevalence in patients with known COPD. A second aim was to explore and identify sources of heterogeneity in PD prevalence estimates. This study therefore proposes to fill several evidence gaps by the following: (1) Applying meta-analysis techniques to provide a precise estimate of PD prevalence in COPD globally and (2) addressing the considerable variation and heterogeneity in prior reviews by applying moderator and meta-regression techniques that might explain variation in PD prevalence.

## Methods

### Search strategy

This study adheres to the Preferred Reporting Items for Systematic reviews and Meta-Analyses (PRISMA checklist in Supplement Digital Content 1) [24] and was prospectively registered on PROSPERO (CRD42024559743). A search of five electronic databases was performed up to January 2025, Embase, PubMed, PsycINFO, Scopus, and Web of Science, using search terms “panic,” “anxiety disorder,” “panic disorder,” or “panic attack” combined using Boolean logic with “chronic obstructive pulmonary disease” (full search strings in Supplement Digital Content 2). A hand search was performed screening the reference lists of prior relevant narrative reviews [15, 17, 19, 20]. After importation of title/abstracts to EndNote, we used the de-duplication process outlined by Bramer et al. [25]. Two reviewers independently screened the title and abstract to assess eligibility (B. T., P. T.). Full text was evaluated if eligibility was not clear from the abstract. At both stages, any disagreements in eligibility were discussed to achieve consensus. For backwards and forward citation searching, we used SpiderCite [26].

### Inclusion criteria

#### Population

Eligible populations were adults age ≥ 18 years with standardized diagnosis of COPD, either through physician diagnosis according to spirometry, international stage criteria (e.g., Global Initiative for Chronic Obstructive Lung Disease [GOLD]), or indicated by International Classification of Disease (ICD) codes in medical records, including any historical versions to allow for changes in obstructive lung disease classification over time. Ineligible populations were those derived from patients receiving lung transplant or self-reported COPD, given that persons with PD may attribute their symptoms to a medical condition in the absence of a verified diagnosis [11, 27].

#### Condition

The condition of interest was PD determined using a standardized and structured clinical interview which is considered the gold standard for determining psychiatric diagnoses. Because panic attacks may be experienced as directly related to another medical condition such as COPD, we also considered eligible studies that attributed panic attacks to COPD using the DSM-IV diagnostic category “anxiety disorder due to a general medical condition” or DSM-5 category “anxiety due to another medical condition” where panic attacks are the dominant clinical feature. In ICD, commensurate diagnoses include organic anxiety disorder and anxiety disorder due to known physiological condition (F06.4). We also considered eligible studies conversely adopting modified diagnostic criteria to include only panic disorder attributable to non-COPD symptoms or studies using blinding where COPD status was unknown to the rater.

#### Comparator

The comparator group was persons with COPD who underwent structured interviews and were without the outcome of interest (PD), with additional comparators derived from case-control studies including general/healthy population samples or other non-COPD medical conditions who underwent standardized and structured clinical interviews to determine the condition of interest.

#### Outcome

The outcome is the proportion of PD calculated by dividing the number of persons with PD by the total number of persons with COPD multiplied by 100 to produce a prevalence rate expressed as a percentage (*n*_PD_/N × 100_COPD_). The prevalence in a case-control comparator is derived in similar fashion, dividing the number of persons with PD by the total number of persons in the comparator COPD multiplied by 100 to produce a prevalence rate expressed as a percentage (*n*_PD_/N × 100_COMPARATOR_).

#### Study design and setting

Eligible studies were longitudinal studies, case-control studies, cross-sectional analytical studies, and database registry or retrospective studies written in the English language. Ineligible studies included case reports, qualitative studies, conference abstracts, and studies with sample sizes of fewer than 20 participants with COPD, such as case series. The minimum sample size was set to permit the inclusion of case-control studies where prior reports indicated substantially high, albeit plausible, estimates of PD.

### Data extraction

Data extraction used a standardized Microsoft Excel^®^ extraction template. Extraction was undertaken by two authors independently (B. T., P. T.), and consensus was achieved by discussion and reviewing each other’s work. Data extracted included the study identifiers (author and year of publication, the country(ies) of recruitment and study region where there was potential sample overlap), general descriptive characteristics (mean age, percentage of males, COPD, and control sample sizes), study characteristics (recruitment from inpatients or general community), COPD characteristics (post-bronchodilator forced expiratory volume in 1 s [FEV₁]/forced vital capacity [FVC] ratio, spirometry predicted FEV₁, proportion of smokers, self-report COPD measures, mean COPD duration) and psychiatric assessment characteristics (interview type, classification system, number of panic disorder cases detected current/past, inclusion of agoraphobia, modification of criteria due to medical condition, rater blinding to COPD status, rater qualifications), and psychiatric comorbidity factors (prevalence of major depression and dysthymia and generalized anxiety disorder and any other anxiety disorder, use of antidepressant medication, use of anxiolytic drugs including benzodiazepines and z-drugs according to Anatomic Therapeutic Chemical classification).

### Study quality

Study quality was assessed independently by two reviewers (B. T., P. T.) according to the Joanna Briggs Institute (JBI) case-control or analytical cross-sectional checklists [28]. Individual items are individually rated as either yes, no, or unclear, and raters are advised to abstain from tallying together different items to produce a score that dilutes the importance of heterogeneous quality domains [28].

### Data analysis

Data was analyzed using Comprehensive Meta-Analysis Version 4 [29], calculating the weighted proportion of participants with a diagnosis of panic disorder (n, numerator) from the number of COPD patients within a study (N, denominator). Where an eligible study performed structured interview but reported no panic disorder cases, we imputed 0.5 cases to the study’s dataset to permit calculation of the standard error and 95% confidence interval (CI) as well as the Bayesian prediction interval (PI). This method is used to limit upward biases that overestimate the prevalence of panic disorder as a result of excluding low prevalence data and thus serves to offset publication biases [30]. Imputation was performed in three studies [31–33]. The presence of publication bias was evaluated with the tests of Egger [34], Begg-Mazumdar [35], Duval and Tweedie [36], and visual inspection of funnel plot asymmetry [37]. A one study removed sensitivity analysis was conducted in combination with visual inspection of forest plots and transformation of prevalence rates to z-scores to identify outliers.

The pooled prevalence of panic disorder in patients with COPD was calculated using a random effects model under the assumption of considerable clinical, methodological, and statistical heterogeneity. The *I*^2^ statistic was used to quantify heterogeneity with values > 60% signifying considerable heterogeneity in prevalence between studies [38]. A pre-planned meta-regression to explore heterogeneity utilized the restricted maximum likelihood assumption and included the intercept. The meta-regression with integer variables shows the unit change in prevalence (as β) per unit change in predictor variable, with associated 95% CI, *p*-value, and goodness of fit. A candidate variable had to be available in ≥ 10 studies for inclusion in the meta-regression. Regression models included a bivariate model with mean age and proportion of male sex and univariate models for spirometry FEV₁/FVC ratio, FEV_1_ predicted, prevalence of current/former smokers, COPD mean duration, prevalence of major depression, and generalized anxiety disorder. An insufficient number of studies reported antidepressant and anxiolytic medication to perform meta-regression. For categorical variables, we performed subgroup analyses for potential moderators and compared groups based on the following: study region, study design, sampling (general community, inpatient, outpatient, mix in- and outpatient settings), subjective COPD measures (yes/no), psychiatric interview type, psychiatric classification system, panic ± agoraphobia specified (yes/no or separate), lifetime panic disorder recorded (yes/no), panic criteria modified to exclude cardiopulmonary symptoms (yes/no) or blinded rater, and rater qualifications.

### Deviations from protocol

A summary of deviations from the original protocol is provided in Supplemental Digital Content 3.

## Results

### Characteristics of included studies

**Fig. 1:**
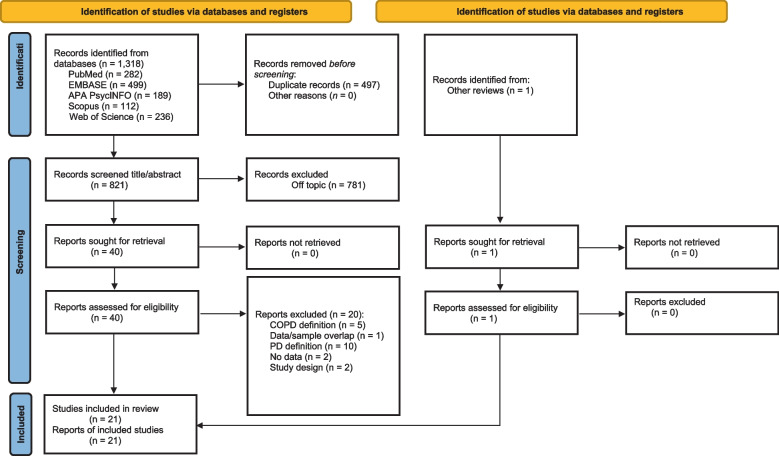
A PRISMA flow chart of the article selection process. A flowchart showing the article selection process for the systematic review from electronic databases and other sources; APA PsycINFO, American Psychiatric Association Psychological Information Database; COPD, chronic obstructive pulmonary disease; Embase, Excerpta Medica dataBASE; NR, not reported; PD, panic disorder; PRISMA, Preferred Reporting Items for Systematic reviews and Meta-Analyses; PubMed, Publisher Medical Literature Analysis and Retrieval System Online

From 1138 articles retrieved during the database search, 40 were screened at full text (Supplement Digital Content 4), and 21 articles were retained for inclusion (Fig. 1). Articles described populations most commonly from Asia (*k* = 9), with other articles representing North America (*k* = 4), Oceania (*k* = 4), Europe (*k* = 3), and Africa (*k* = 1, Table 1). The studies reported on 1847 persons with COPD. The median proportion of males was 69 ± 19%, and the mean age of participants was 63 ± 7 years. Among the 9 studies utilizing control groups, the median healthy control sample size was 103 persons (cumulative *N* = 1008 healthy controls), and the median chronic disease sample size was 30 (cumulative *N* = 450 medical controls). The median forced expiratory volume in 1 s (FEV₁)/forced vital capacity (FVC) ratio in eight studies was 0.55, and the median FEV_1_ predicted was 49.7% (Table 2). Diagnosis of PD was most commonly adjudicated by psychiatrists (*k* = 6), followed by psychologists (*k* = 3) and physicians (*k* = 2) (Supplement Digital Content 5). The most common diagnostic criteria used was DSM-IV (*k* = 10), followed by DSM-5 (*k* = 5) and ICD-10 (*k* = 4). Comorbid major depressive disorder and comorbid generalized anxiety disorder were reported in *k* = 12 studies with a median of 14% and 6% prevalence respectively (Supplement Digital Content 6).

**Table 1:** General descriptive characteristics of included studies reporting panic disorder in COPD

Study ID	Country^a^	Design	Setting	COPD cases	% male	Mean age, years	Comparator	Matching
Aghanwa [31]	Nigeria	Case control	In and outpatients	30	83	62.9	Hypertension (*n* = 30) Healthy control (*n* = 30)	None
Aydin [32]	Turkey	Case control	Inpatients	38	100	48	Tuberculosis (*n* = 119)	None
Baker [39]	USA (multi-state)	Cross-sectional	General	219	54	65	-	-
Chandel [33]^b^	India (Kathua, Jammu, and Kashmir)	Cross-sectional	Inpatients	50	82	58	-	-
Chaudhary [40]	India (Lucknow, Uttar Pradesh)	Case control	Outpatients	74	55	77	Healthy control (*n* = 74)	Age-sex
Dar [41]^b^	India (Srinagar, Jammu, and Kashmir)	Case control	UC	100	50	56.2	Healthy control (*n* = 100)	Age-sex
Dowson [22]	New Zealand	Cohort study	Inpatients	76	37	65.3	-	-
Karajgi [42]	USA (Queens, New York)	Cross-sectional	Outpatients	50	62	64.9	-	-
Kuhl [43]	Germany (Marburg, Hesse)	Case control	Outpatients	143	78	67.9	Healthy control (*n* = 105)	Spouse
Kunik [44]	USA (Houston, Texas)	Cross-sectional	General	204	96	65.9	-	-
Laurin [45]	Canada	Cross-sectional	Outpatients	116	47	67	-	-
Livermore [14]^c^	Australia (Sydney, New South Wales)	Case control	Outpatients	52	45	72.6	Healthy controls (*n* = 20)	Age
Livermore [6]^c^	Australia (Sydney, New South Wales)	Cross-sectional	Outpatients	62	44	72.9	-	-
Mehta [46]	India (Bhavnagar, Gujarat)	Cross-sectional	Outpatients	59	92	NR	-	-
Pascal [47]	Romania	Cross-sectional	Outpatients	60	87	62.2	-	-
Pothirat [48]^d^	Thailand	Cross-sectional	General	82	73	54.9	Healthy (*n* = 228) Other respiratory (*n* = 216)	None
Sharma [49]^e^	India (Bareilly, Uttar Pradesh)	Cross-sectional	In and outpatients	140	86	56.8	-	-
Singh [50]	India (Pune, Maharashtra)	Cross-sectional	In and outpatients	122	64	53.9	Healthy (*n* = 122)	Age-sex
Sood [51]	India (Shimla, Himachal Pradesh)	Cross-sectional	Outpatients	100	73	64.3	-	-
Vogele [52]	Germany (Greifswald, Mecklenburg-Western Pomerania)	Case control	Inpatients	20	70	62.2	Orthopedic inpatients (*n* = 20)	None
Yellowlees [53]	Australia (Adelaide, South Australia)	Cross-sectional	Inpatients	50	64	65	-	-

*COPD *chronic obstructive pulmonary disease.

^a^For countries where multiple studies were eligible, we describe the study region and province to demonstrate non-overlapping samples.

^b^The studies from Jammu and Kashmir regions were adjudicated as independent samples because they were recruited from different hospitals with nonoverlapping dates 2022 [33] and 2015–2016 [41].

^c^There was concern that the two Livermore studies [6, 14] were derived from the same sample, with both recruiting from 96 consecutive outpatients approached; however, one reported a case-control design, and the panic disorder prevalence rate was markedly different between studies (9/52 = 17.3% c.f. 25/62 = 40.3%).

^d^Pothirat (2015) [48] was a cross-sectional design but provided subgroup data for non-respiratory cases and was analyzed in the meta-analysis as a case-control study.

^e^The Sharma study [49] reported 17.3% prevalence in text. The number of people with panic disorder was calculated from Table 1 as 27/140 = 19.3%.

**Table 2:** Study characteristics relating to chronic obstructive pulmonary disease

Study ID	Spirometry FEV₁/FVC ratio	FEV_1_ predicted %	Current or former smoker %	Self-report COPD severity	COPD duration years
**Median**	**0.55**	**49.7**	**72**	**-**	**6.9**
Aghanwa [31]	NR	NR	NR	No	> 2
Aydin [32]	NR	NR	NR	No	NR
Baker [39]	0.51	49.7	98	CAT	9
Chandel [33]	NR	NR	30	No	NR
Chaudhary [40]	0.57	38	72	No	5
Dar [41]	0.55	38	73	No	NR
Dowson [22]	NR	41	40	No	NR
Karajgi [42]	NR	NR	NR	No	NR
Kuhl [43]	NR	50	73	No	NR
Kunik [44]	NR	NR	NR	No	NR
Laurin [45]^a^	0.54	42	50	SGRQ	9
Livermore [14]	NR	54	NR	No	6.8
Livermore [6]	0.52	53	90	No	6.6
Mehta [46]^b^	0.60	46	41	CAT, mMRC	-
Pascal [47]	NR	NR	98	CAT, mMRC	1
Pothirat [48]	53	50	NR	ECRHS	NR
Sharma [49]	NR	NR	63	No	7
Singh [50]	NR	NR	56	CAT, SGRQ	NR
Sood [51]	NR	NR	NR	No	NR
Vogele [52]	0.61	75	NR	No	14.1
Yellowlees [53]	NR	NR	80	No	NR

*CAT *COPD Assessment Test, *COPD *chronic obstructive pulmonary disease, *ECRHS *European Community Respiratory Health Survey, *FEV₁/FVC* ratio forced expiratory volume_1_/forced vital capacity, *mMRC *modified Medical Research Council Dyspnea Scale, *NR *not reported, *SGRQ *St. George Respiratory Questionnaire, *TB *tuberculosis.

^a^Current smoker only [45].

^b^The M(SD) data for duration of COPD was comparable to the age of patients and deemed implausible and thus not reported here [46].

### Quality assessment

In the *k* = 8 case-control studies, study quality was generally favorable with 75–88% of studies meeting quality criteria for the exposure being valid, measured appropriately, the outcome being reliable and valid, and appropriate statistics being used (Supplement Digital Content 7 & 8). Items where quality was lower or unclear pertained to group comparability (38% of studies) and case-control matching (63% of studies). In the *k* = 13 cross-sectional analytical studies, study quality was generally favorable with study quality adjudicated as sufficient for most items, being lowest (85%) for inclusion criteria being clearly defined and highest (92%) for all other items.

### Data synthesis and meta-analysis

**Fig. 2:**
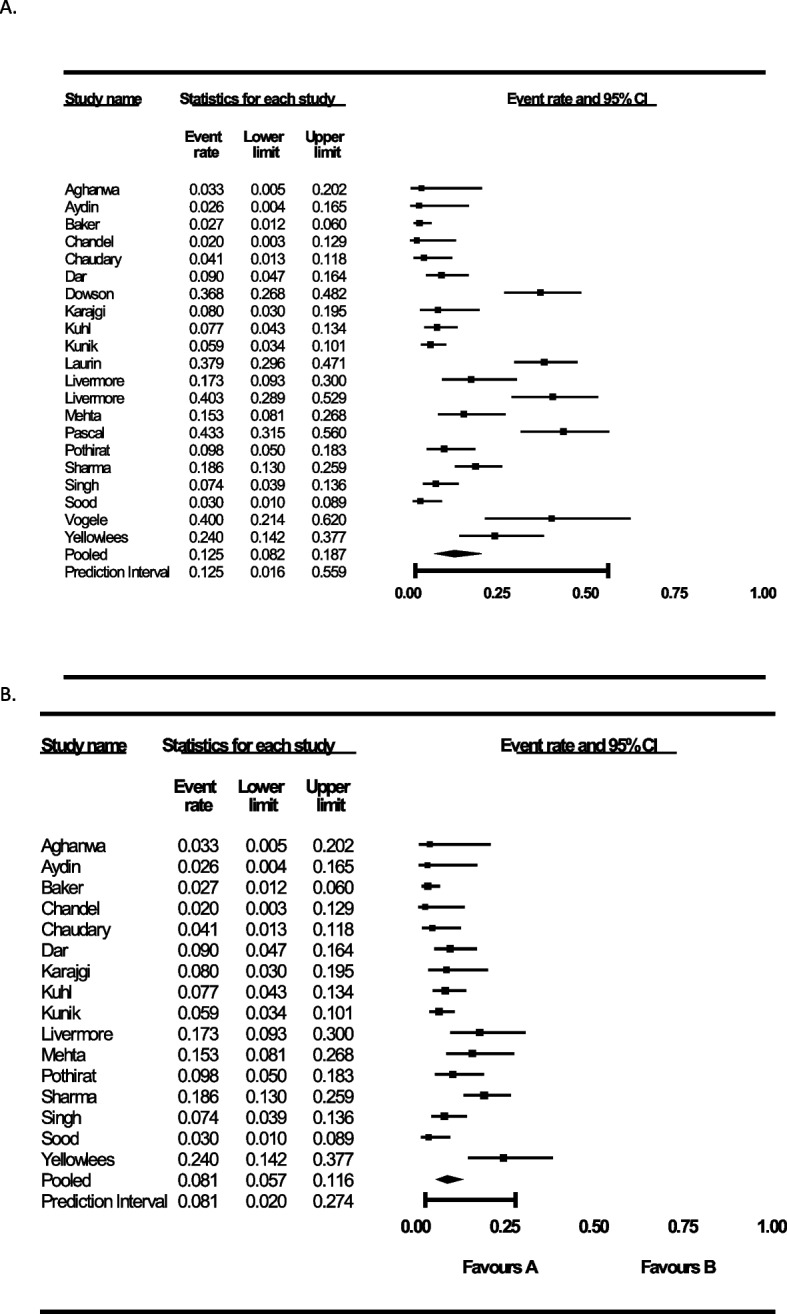
Forest plot showing the event rate of panic disorder in populations with COPD with outliers (**A**) and without outliers (**B**). The forest plot shows the crude prevalence rate of panic disorder in persons with COPD per study and the 95% confidence interval (CI). All studies were combined together using the inverse variance method with random-effects and is denoted as “pooled.” The prediction interval is the Bayesian posterior estimate of panic disorder prevalence in similar COPD populations that would be expected in 95% of replicates. Prevalence rates in text are reported as proportion n/N and can be multiplied by 100 to produce a prevalence in percent

**Fig. 3:**
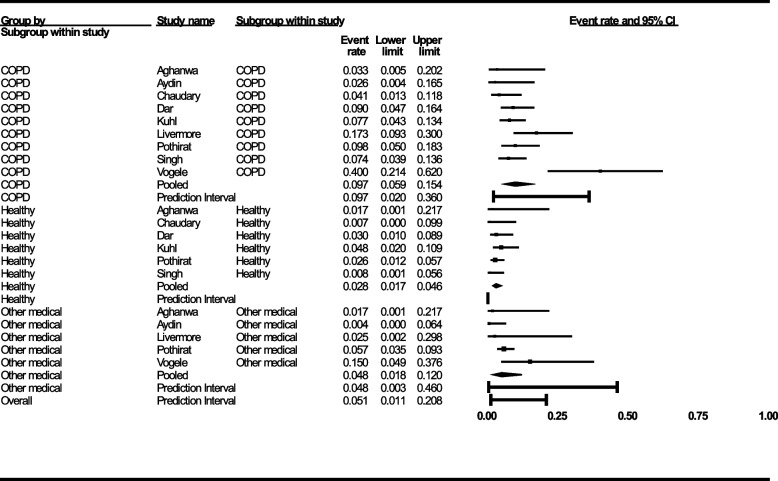
Forest plot showing the event rate of panic disorder in populations with COPD by comparison to healthy controls and persons with other medical conditions. The forest plot shows the crude prevalence rate of panic disorder in persons with COPD per study and the 95% confidence interval (CI). All studies were combined together using the inverse variance method with random effects and is denoted as “pooled.” The prediction interval is the Bayesian posterior estimate of panic disorder prevalence in similar populations that would be expected in 95% of replicates. Prevalence rates are reported as proportion n/N and can be multiplied by 100 to produce a prevalence in percent

The pooled PD prevalence from *k* = 21 studies was 12.5% (95% CI 8.2–18.7) with considerable heterogeneity (*I*^2^ = 90%) and a wide 95% Bayesian prediction interval ([PI] 1.6–55.9) (Fig. 2, Supplement Digital Content 9). Visual inspection of the funnel plot (Supplement Digital Content 10) and publication bias metrics (Supplement Digital Content 11) suggest publication bias. The trim-and-fill imputation of four studies to the right of the mean resulted in an increased PD prevalence estimate of 15.9% (CI, 9.8–24.6), suggesting the true prevalence may be higher than the pooled estimate of published results. Effect estimates were higher in COPD (*k* = 9, 9.7%; 95% CI 5.9–15.4, *I*^2^ = 69) than healthy controls (*k* = 6, 2.8%; 95% CI 1.7–4.6, *I*^2^ = 0), but not other medical conditions (*k* = 5, 4.8%; 95% CI 1.8–12.0, *I*^2^ = 47, *p* < 0.01 for between-group difference) (Fig. 3).

Visual inspection of the forest plot and z-scores, in combination with the one study removed sensitivity analysis (Supplement Digital Content 12), identified *k* = 5 studies as outliers [6, 22, 45, 47, 52], and we present results with and without outliers herein [37]. After removal of outliers, the analysis with *k* = 16 effect sizes revised the pooled prevalence to 8.1% (95% CI 5.7–11.6, *I*^2^ = 73) with a 95% PI 2.0–27.4. There was also evidence to suggest publication bias, with the trim-and-fill imputation of five studies to the right of the mean resulting in an increased prevalence estimate of 10.6% (95% CI 7.4–15.0). 

### Meta-regression and subgroup moderators

The meta-regression did not reveal any change in PD prevalence attributable to the continuous predictor variables (Supplement Digital Content 13). Among the categorical variables, the study region was identified as a significant moderator in the main and outlier removed analysis with higher estimates observed in Oceania than Asia (Supplement Digital Content 14). Psychiatric interview type was also a significant moderator in the main and outlier removed analysis. In the main analysis, prevalence estimates obtained from the Anxiety Disorder Interview Schedule (ADIS, [54]) and the Diagnostisches Interview für Psychische Störungen-Forschungsversion (DIPS, [55]) were higher and nonoverlapping with 95% CI estimates obtained from all other measures. Rater qualifications were also observed as a significant moderator in the main and outlier removed analysis, with clinical psychologist qualifications associated with higher PD prevalence than other professionals. Conversely, in outlier removed analyses, psychiatrist raters yielded higher prevalence estimates.

## Discussion

This review estimated a PD prevalence in COPD between 8 and 12.5%, with a higher prevalence observed when PD rates were compared to healthy control groups but not when compared to persons with other medical conditions. When outliers were excluded, the prevalence estimate was 8.1% though the true prevalence could be higher given the evidence for publication bias and wide Bayesian 95% PIs. Nonetheless, the pooled prevalence estimates and PIs here all fell within the lower ranges reported in previous narrative reviews and suggest that prior reports up to ~50% PD prevalence in COPD are overestimated.

Here, the observed estimates for PD in COPD exceeded the healthy control estimate of 2.8% (95% CI 1.7–4.6), as well as the lifetime PD prevalence reported in the World Mental Health surveys of 0.8% in low- and 2.2% in high-income countries, respectively [56]. Common mechanisms are purported to underlie PD in both COPD and non-COPD populations. Fear of physical sensations of anxiety (i.e., anxiety sensitivity) has been demonstrated in COPD patients with PD [6, 14, 57], corroborating foundational research in the general PD population without medical comorbidities [58]. Moreover, heightened sensitivity to physical symptoms is associated with COPD exacerbations and respiratory-related hospitalizations [13] as well as poorer exercise tolerance in COPD [57]. It is possible that the threshold for perception of bodily sensations or amplification of neutral autonomic arousal stimuli is lowered by the prospect of genuine threat such as COPD exacerbation. Here, the studies which modified diagnostic criteria, using an exclusive approach to ensure cardiopulmonary symptoms, were not attributed to PD and did not moderate the prevalence rate. Diagnostic approaches that include somatic symptoms in depression diagnoses are considered optimal in chronic disease populations [59]. In COPD, PD patients report more cardiovascular and gastrointestinal symptoms than their non-PD counterparts [52]. COPD patients also report experiencing air hunger, smothering, sensations of choking, and suffocation [4]. It is possible that there are discrete subtypes of PD in COPD. It was suggested that non-fearful variants of PD are more common in cardiopulmonary populations than fearful PD and agoraphobia [60–62]. Specifically, non-fearful variants are atypical and characterized by an absence of fear of dying, impending catastrophe, or going crazy but still characterized by physical symptoms.

Changing nomenclature, diagnostic criteria, and clinical conceptualizations of PD and agoraphobia could explain some of the variations in prevalence rates over time [56]. Only one study used DSM-5 criteria to report agoraphobia as a distinct disorder, and only five studies reported agoraphobia alongside PD, underscoring the need for further empirical research to quantify PD in relation to agoraphobia in cardiopulmonary diseases. Reporting current and lifetime disorder history for PD with or without agoraphobia would help elucidate whether these disorders develop primarily after the onset of COPD. In heart failure populations, the prevalence of current PD onset prior to heart failure was similar to the incident rate which developed after heart failure, and the latter had longer hospital admissions [63].

There was evidence that the diagnostic interview type (ADIS *k* = 3, FDIPS *k* = 1) and qualifications of the reviewer (psychologist *k* = 4) were associated with higher PD prevalence estimates in the main analysis. Notably, these two methodological characteristics were overlapping within *k* = 3 studies [6, 45, 52] which involved the ADIS or FDIPS as well as psychologist raters. The ADIS was found to yield similar PD prevalence in asthma and non-asthma populations [64] but a relatively higher proportion of PD in persons with unexplained chest pain [65] and noncardiac chest pain [66] than that reported here and in past heart disease studies [30]. This suggests that the ADIS may be particularly sensitive towards detecting or including cardiopulmonary symptoms as part of PD. The finding of higher PD prevalence in psychologists was not corroborated in outlier removed analyses where psychiatrist raters yielded higher PD estimates. The latter outlier removed finding is consistent with a previous review of PD prevalence in coronary heart disease, where PD prevalence estimates were higher among psychiatric trained raters (9.92% in heart disease) than nonpsychiatric trained raters (4.74% in heart disease) [30]. The consistent findings across heart disease and COPD might indicate that psychiatrists’ comprehensive training in medicine and psychiatry enhances diagnostic detection [67], using an inclusion approach to somatic symptoms while taking into consideration diagnostic exclusion rules [59].

The clinical implications of this review include that PD prevalence here was much lower than what is promulgated in prior narrative reviews. Clinicians in respiratory care, cardiopulmonary rehabilitation, and consultation-liaison psychiatry and psychology should expect to observe between 8 and 12.5% PD prevalence in COPD. The low PD prevalence estimates here by comparison to prior narrative reviews have potential implications for triaging patients. The presence of a known mental health condition such as PD can lead to under-recognition and disregard of physical symptoms, known as diagnostic overshadowing [68]. Expectations for considerably high and diagnostically questionable PD prevalence rates in COPD patients may result in unnecessary disregard of patient symptoms and their concerns.

Other clinical implications relate to the treatment of co-occurring panic attacks in cardiopulmonary diseases. Hyperventilation, breathing through a narrow straw, and breath holding are among the most common respiratory exposure techniques utilized in PD treatment in the absence of COPD or other medical conditions [69, 70]. Interoceptive exposure techniques for anxiety disorders in comorbid medical conditions are less well characterized [71, 72] and may pose some risk to cardiopulmonary patients [73]. Moreover, overt and covert avoidances of exercise, cardiopulmonary rehabilitation, and exposure therapy due to fear of exacerbating the condition are common [74, 75], pointing to unique challenges when treating PD in COPD. Past experimental and observational studies generally suggest PD is not associated with impaired indices of cardiopulmonary function per se [14, 18, 76–79]. Rather, increased vigilance to autonomic symptoms and negative cognitive-affective interpretations of cardiopulmonary symptoms seem to play a central role in magnifying the intensity of symptoms before and during a panic attack in cognitive-behavioral models [15, 22, 52, 71]. However, such models only characterize the predominant fearful panic variant and not the atypical non-fearful variants. There is therefore a need to further investigate the cognitive-affective components of PD in COPD populations and combine this research with contemporary conceptualizations of dyspnea [80, 81]. Cross-sectional and experimental designs could help elucidate key mechanisms and refine intervention approaches for PD in COPD populations.

### Study strengths and limitations

A strength of this study was its inclusion of only studies utilizing structured diagnostic interview to determine the presence or absence of PD. The findings are presented with several limitations which limit the conclusions that can be drawn from the present review. There was under-representation of studies from geographical areas outside Asia limiting the generalizability to Africa (*k* = 1) and South America (no studies). Geographic region was identified as a source of heterogeneity with higher PD prevalence found in Oceania in comparison to Asia. COPD rates are known to differ across regions, as does the contribution of smoking and air pollution to COPD [2]. COPD prevalence globally is highest in the USA, UK, and India — all countries from which most of the eligible studies were recruited [82]. Consequently, the pooled estimates here may be overestimated. Low- and middle-income countries account for the largest increase in disability-adjusted life years and COPD burden with risk factors including smoking, secondhand smoke, household air pollution from solid fuels, ambient particulate matter pollution, occupational particulate matter, gases, and fumes [83]. There was also evidence to suggest publication bias though estimates with the trim-and-fill method were higher following imputation. This result is potentially explained by our exclusion of conference abstracts, gray literature, and unpublished research (all of which may lead to upward bias in prevalence estimates), as well as studies using nonstructured interview estimates (e.g., medical records). Likewise, only studies published in English were retained which can result in articles being omitted from non-English-speaking countries and lower- and middle-income economies. The overrepresentation of studies from Western, educated, industrial, rich, and democratic societies [84] may lead to an underestimation of PD prevalence. Though we performed a hand search and backward/forward citation, it is possible that eligible studies were missed during the electronic database search.

A limitation of the literature was the small sample sizes of included studies, which ranged from 20 to 204 persons (median 74). The use of structured psychiatric interviews can limit the feasibility of performing larger powered studies to determine accurate prevalence estimates. However, the cumulative sample of 1847 participants with COPD was smaller than the 4713 pooled sample reported for PD and coronary heart disease [30]. This discrepancy suggests that more studies with larger samples adopting structured psychiatric interviews are required to improve our understanding of PD in COPD. Furthermore, the small sample sizes in retained articles likely contribute to the variation in prevalence estimates and PI observed here, as does the use of imputation and inclusion of articles where information was unclear when rating study quality.

## Conclusions

In conclusion, this review found between 8 and 12.5% PD prevalence in persons with COPD. The PD prevalence was higher among people with COPD than their healthy control group counterparts. This review highlights a need for further case-control research in COPD populations that employ structured psychiatric interviews in diverse geographic regions to reliably quantify the prevalence of PD and agoraphobia. Doing so may, in turn, prompt further investigation to build knowledge and offer insights into possible cognitive-behavioral mechanisms underlying PD in COPD as well as their impact on emergency department visits and COPD exacerbations. Given the relationship between PD and adverse COPD outcomes [3], there is also a need to adapt and refine treatments and examine their impact on healthcare resource utilization.

## Supplementary Information

Additional file 1

## Data Availability

All data generated and analyzed required to replicate this review, including search strings, is reported in tables, figures, and supplement.

## Abbreviations

CI: Confidence interval
COPD: Chronic obstructive pulmonary disease
DSM: *Diagnostic and Statistical Manual of Mental Disorders*
GOLD: Global Initiative for Chronic Obstructive Lung Disease
ICD: International Classification of Disease
JBI: Joanna Briggs Institute
FEV₁: Forced expiratory volume in 1 s
FVC: Forced vital capacity
PI: Prediction interval
PD: Panic disorder
