# Supplementary material for: The prevalence of panic disorder in chronic obstructive pulmonary disease: a systematic review, meta-analysis, and meta-regression
Source: Syst Rev. 2026 Feb 16;15:93. doi: 10.1186/s13643-026-03102-3 (PMC13014743; doi:10.1186/s13643-026-03102-3)
Supplement: Supplementary file 1 — Additional file 1 [file 13643_2026_3102_MOESM1_ESM.docx]

# **Supplemental Digital Content for The Prevalence of Panic Disorder in COPD: A Systematic Review, Meta-Analysis and Meta-Regression**

**Supplement 1. PRISMA checklist**

**Supplement 2. Search Strings**

**Supplement 3. Deviations from review Protocol (CRD42024559743)**

**Supplement 4. Reasons for exclusion after inspection of full text**

**Supplement 5. Study characteristics pertaining to psychiatric classification and ratings**

**Supplement 6. Psychiatric prevalence data for other conditions and medications**

**Supplement 7. Adjudication of case-control studies according to JBI checklist**

**Supplement 8. Adjudication of study quality in cross-sectional analytical studies according to JBI checklist**

**Supplement 9. Bayesian prediction intervals for panic disorder prevalence in COPD**

**Supplement 10. Funnel plots to ascertain publication bias**

**Supplement 11.** **Tests of publication bias for the prevalence of panic disorder in COPD**

**Supplement 12.** **Forest plots showing the change in panic disorder prevalence when one study was removed from analysis**

**Supplement 13. Meta-regression and moderator analysis for continuous variables**

**Supplement 14. Sub-group moderator analyses for categorical variables**

**Supplement 15. References**

**Supplement 1. PRISMA checklist**

| **Section and Topic** | **Item #** | **Checklist item** | **Location where item is reported** |
| --- | --- | --- | --- |
| **TITLE** | | |  |
| Title | 1 | Identify the report as a systematic review. | Title |
| **ABSTRACT** | | |  |
| Abstract | 2 | See the PRISMA 2020 for Abstracts checklist. | 3-4 |
| **INTRODUCTION** | | |  |
| Rationale | 3 | Describe the rationale for the review in the context of existing knowledge. | 6-7 |
| Objectives | 4 | Provide an explicit statement of the objective(s) or question(s) the review addresses. | 7 |
| **METHODS** | | |  |
| Eligibility criteria | 5 | Specify the inclusion and exclusion criteria for the review and how studies were grouped for the syntheses. | 7-9 |
| Information sources | 6 | Specify all databases, registers, websites, organisations, reference lists and other sources searched or consulted to identify studies. Specify the date when each source was last searched or consulted. | 7 |
| Search strategy | 7 | Present the full search strategies for all databases, registers and websites, including any filters and limits used. | S2 |
| Selection process | 8 | Specify the methods used to decide whether a study met the inclusion criteria of the review, including how many reviewers screened each record and each report retrieved, whether they worked independently, and if applicable, details of automation tools used in the process. | 7 |
| Data collection process | 9 | Specify the methods used to collect data from reports, including how many reviewers collected data from each report, whether they worked independently, any processes for obtaining or confirming data from study investigators, and if applicable, details of automation tools used in the process. | 9-10 |
| Data items | 10a | List and define all outcomes for which data were sought. Specify whether all results that were compatible with each outcome domain in each study were sought (e.g. for all measures, time points, analyses), and if not, the methods used to decide which results to collect. | 9-10 |
|  | 10b | List and define all other variables for which data were sought (e.g. participant and intervention characteristics, funding sources). Describe any assumptions made about any missing or unclear information. | 9-10 |
| Study risk of bias assessment | 11 | Specify the methods used to assess risk of bias in the included studies, including details of the tool(s) used, how many reviewers assessed each study and whether they worked independently, and if applicable, details of automation tools used in the process. | 10 |
| Effect measures | 12 | Specify for each outcome the effect measure(s) (e.g. risk ratio, mean difference) used in the synthesis or presentation of results. | 9 |
| Synthesis methods | 13a | Describe the processes used to decide which studies were eligible for each synthesis (e.g. tabulating the study intervention characteristics and comparing against the planned groups for each synthesis (item #5)). | 8-11 |
|  | 13b | Describe any methods required to prepare the data for presentation or synthesis, such as handling of missing summary statistics, or data conversions. | 10-11 |
|  | 13c | Describe any methods used to tabulate or visually display results of individual studies and syntheses. | 10-11 |
|  | 13d | Describe any methods used to synthesize results and provide a rationale for the choice(s). If meta-analysis was performed, describe the model(s), method(s) to identify the presence and extent of statistical heterogeneity, and software package(s) used. | 10-11 |
|  | 13e | Describe any methods used to explore possible causes of heterogeneity among study results (e.g. subgroup analysis, meta-regression). | 11 |
|  | 13f | Describe any sensitivity analyses conducted to assess robustness of the synthesized results. | 11 |
| Reporting bias assessment | 14 | Describe any methods used to assess risk of bias due to missing results in a synthesis (arising from reporting biases). | NA |
| Certainty assessment | 15 | Describe any methods used to assess certainty (or confidence) in the body of evidence for an outcome. | NA |
| **RESULTS** | | |  |
| Study selection | 16a | Describe the results of the search and selection process, from the number of records identified in the search to the number of studies included in the review, ideally using a flow diagram. | 12, Fig 1 |
|  | 16b | Cite studies that might appear to meet the inclusion criteria, but which were excluded, and explain why they were excluded. | S4 |
| Study characteristics | 17 | Cite each included study and present its characteristics. | Table 1, Table 2, S5, S6 |
| Risk of bias in studies | 18 | Present assessments of risk of bias for each included study. | 12-13, S7, S8 |
| Results of individual studies | 19 | For all outcomes, present, for each study: (a) summary statistics for each group (where appropriate) and (b) an effect estimate and its precision (e.g. confidence/credible interval), ideally using structured tables or plots. | 13, Fig 2, Fig 3 |
| Results of syntheses | 20a | For each synthesis, briefly summarise the characteristics and risk of bias among contributing studies. | 12-13, S6, S7 |
|  | 20b | Present results of all statistical syntheses conducted. If meta-analysis was done, present for each the summary estimate and its precision (e.g. confidence/credible interval) and measures of statistical heterogeneity. If comparing groups, describe the direction of the effect. | 12-13, Fig 2, Fig 3 |
|  | 20c | Present results of all investigations of possible causes of heterogeneity among study results. | 12-13, S12, S13 |
|  | 20d | Present results of all sensitivity analyses conducted to assess the robustness of the synthesized results. | 13, Fig 2, Fig 3, S12, S13 |
| Reporting biases | 21 | Present assessments of risk of bias due to missing results (arising from reporting biases) for each synthesis assessed. | NA |
| Certainty of evidence | 22 | Present assessments of certainty (or confidence) in the body of evidence for each outcome assessed. | NA |
| **DISCUSSION** | | |  |
| Discussion | 23a | Provide a general interpretation of the results in the context of other evidence. | 14 |
|  | 23b | Discuss any limitations of the evidence included in the review. | 15-18 |
|  | 23c | Discuss any limitations of the review processes used. | 15-18 |
|  | 23d | Discuss implications of the results for practice, policy, and future research. | 15-18 |
| **OTHER INFORMATION** | | |  |
| Registration and protocol | 24a | Provide registration information for the review, including register name and registration number, or state that the review was not registered. | 3, 7 |
|  | 24b | Indicate where the review protocol can be accessed, or state that a protocol was not prepared. | 3, 7, S3 |
|  | 24c | Describe and explain any amendments to information provided at registration or in the protocol. | S3 |
| Support | 25 | Describe sources of financial or non-financial support for the review, and the role of the funders or sponsors in the review. | Footnotes to the manuscript |
| Competing interests | 26 | Declare any competing interests of review authors. | Footnotes the manuscript |
| Availability of data, code and other materials | 27 | Report which of the following are publicly available and where they can be found: template data collection forms; data extracted from included studies; data used for all analyses; analytic code; any other materials used in the review. | Table 1, Table 2, S5, S6 |

*Adapted from Page et al.^1^*

*PRISMA, Preferred Reporting Items for Systematic Reviews and Meta-Analyses;*

**Supplement 2. Search Strings**

Search performed January 23^rd^ 2025

**Embase**

(panic:ab,ti OR 'anxiety disorder*':ab,ti OR 'panic disorder':ab,ti OR 'panic attack*':ab,ti OR agoraphobia:ab,ti) AND (copd:ab,ti OR 'chronic obstructive pulmonary disease':ab,ti OR 'respiratory disease':ab,ti OR 'respiratory illness':ab,ti OR 'cardio-respiratory condition':ab,ti OR 'cardio-respiratory disease':ab,ti OR 'chronic obstructive lung disease':ab,ti OR coad:ab,ti OR 'chronic obstructive airway disease':ab,ti OR 'airflow obstruction':ab,ti)

# **PsycINFO**

# Panic OR “anxiety disorder*” OR “panic disorder” OR “panic attack*” OR agoraphobia AND COPD OR “chronic obstructive pulmonary disease” OR “respiratory disease” OR “respiratory illness” OR “cardio-respiratory condition” OR “cardio-respiratory disease” OR “chronic obstructive lung disease” OR COAD OR “chronic obstructive airway disease” OR “airflow obstruction”.

# **PubMed**

Panic [tiab] OR “anxiety disorder*” [tiab] OR “panic disorder” [tiab] OR “panic attack*” [tiab] OR agoraphobia [tiab] AND COPD [tiab] OR “chronic obstructive pulmonary disease” [tiab] OR “respiratory disease” [tiab] OR “respiratory illness” [tiab] OR “cardio- respiratory condition” [tiab] OR “cardio-respiratory disease” [tiab] OR “chronic obstructive lung disease” [tiab] OR COAD [tiab] OR “chronic obstructive airway disease” [tiab] OR “airflow obstruction” [tiab]

# **Scopus**

title-abs-key panic{anxiety disorder*}OR {panic disorder}OR{panic attack*}OR agoraphobia AND copd{chronic obstructive pulmonary disease}{respiratory disease}OR{respiratory illness}OR{cardiorespiratory condition}OR{cardiorespiratory disease}OR^2^OR coad{chronic obstructive airway disease}OR{airflow obstruction}

# **Web of Science**

(AB= (Panic OR “anxiety disorder*” OR “panic disorder” OR “panic attack*” OR agoraphobia)) AND AB= (COPD OR “chronic obstructive pulmonary disease” OR “respiratory disease” OR “respiratory illness” OR “cardio-respiratory condition” OR “cardio- respiratory disease” OR “chronic obstructive lung disease” OR COAD OR “chronic obstructive airway disease” OR “airflow obstruction”)

**Supplement 3. Deviations from review Protocol (CRD42024559743)**

| **Section** | **Original** | **Change** | **Justification/rationale** |
| --- | --- | --- | --- |
| **Exposure / Condition** | *or using medical records reflecting a past clinical diagnosis of PD* | *The condition of interest was PD determined using a standardized and structured clinical interview which is considered the gold-standard for determining psychiatric diagnoses.* | We restricted the condition to structured diagnostic interviews because this is the gold-standard for making psychiatric diagnoses, and secondly, this allows a comparison between subgroups based on qualifications that was identified as a source of heterogeneity in a previous review (1). |
| **Comparator** | *The comparator group consists of adults with COPD who do not have a diagnosis of panic disorder.* | *…. comparators derived from case-control studies including general/healthy population samples or other non-COPD medical conditions who underwent standardized and structured clinical interview to determine the condition of interest.* | Rewording the comparator eligibility helped clarify how comparators were selected from case-control studies, permitting comparison between prevalence rates in COPD and non-COPD samples. |
| **Study design and setting (context)** | *Eligibility: All included studies must be written in English and include at least 100 participants.* | Ineligibility: *and studies with sample sizes of fewer than 20 participants such as case-series.* | Reducing the eligible sample size and clarifying these were with COPD helped clarify that case-control studies were eligible and allowed comparisons in PD prevalence between COPD and other conditions to permit the inclusion of case control studies where prior reports indicated substantially high, albeit plausible, estimates of PD. |
| **Search Strings** | *The literature search will include studies completed in English using the electronic databases PubMed, MEDLINE, Scopus, Web of Science, Embase and PsycINFO. An additional hand search of the reference lists of selected articles will be conducted. The search terms ‘panic’ and ‘panic disorder’ will be used in conjunction with the terms ‘COPD’, ‘cardiopulmonary’, ‘cardiorespiratory’, ‘respiratory’, ‘medical illness’ and ‘medical condition’.* | *A search of five electronic databases was performed to January 2025 on; Embase, PubMed, PsycINFO, Scopus, and Web of Science using search terms ‘panic’, ‘anxiety disorder’, ‘panic disorder’, or ‘panic attack’ combined using Boolean logic with ‘chronic obstructive pulmonary disease’* | Consultation with an information retrieval specialist helped clarify the search scope and terms pertaining to the research question, omitting extraneous terms ‘medical illness’ and ‘medical condition.’ The term ‘anxiety disorder’ and ‘agoraphobia’ were added to help identify studies with standardised diagnoses. MEDLINE removed due to being redundant with PubMed search. |

COPD, chronic obstructive pulmonary disease; PD, panic disorder;

<https://www.crd.york.ac.uk/PROSPERO/view/CRD42024559743>

**Supplement 4. Reasons for exclusion after inspection of full text**

| **ID** | **Country** | **Justification** | **Reason** |
| --- | --- | --- | --- |
| Chan ^21^ | Singapore | The PRIME-MD was used as a self-report screener and not structured interview or other diagnosis. | PD definition ineligible |
| Chetty ^22^ | United Kingdom | Data was gathered from primary care data obtained via the National Health Service but panic disorder was not reported, only ‘anxiety and neurotic disorders’ | PD definition ineligible |
| Dua 2018 ^23^ | India | The authors stated a diagnosis was made according to ICD-10 but panic disorder was not reported, only ‘anxiety disorders’ | PD definition ineligible |
| El-Gabalawy ^24^ | Canada | Definition of lung disease not reported, data also reported as odds ratios for any anxiety disorder. | COPD definition ineligible |
| Garg ^25^ | India | The authors stated “diagnoses were made according to ICD-10” but not structured interview; the self-report measure (SCL-80) was used to define panic and phobia etc. | PD definition ineligible |
| Giardino ^26^ | USA | Experimental design incompatible with determining PD prevalence. | Ineligible study design |
| Holas ^27^ | Poland | Anxiety symptoms were measured by self-report questionnaires and not structured interview or other diagnosis. | PD definition ineligible |
| Howard ^28^ | UK | Anxiety symptoms were measured by self-report questionnaires and not structured interview or other diagnosis. | PD definition ineligible |
| Hsieh ^29^ | Taiwan | An ‘any anxiety disorder’ category was used. | PD definition ineligible |
| Livermore ^30^ | Australia | Panic attack incidence, not prevalence (baseline PD excluded from study). | PD definition ineligible |
| Moore ^31^ | USA | The sample was derived from people with breathing problems and not COPD. | COPD definition ineligible |
| Moretta ^32^ | Italy | Narrative review paper with no data. | Ineligible study design |
| Ohayon ^33^ | Germany, Spain, UK | The COPD variable was determined by telephone interview self-report. | COPD definition ineligible |
| Perna ^34^ | Italy | The prevalence of respiratory conditions was reported in persons with PD. | Prevalence not reported |
| Pollak ^35^ | USA | Only a subset of participants underwent structured psychiatric interview that was obtained from a mixed pulmonary function group and data was not reported for all persons with COPD. | COPD definition ineligible |
| Porzelius ^36^ | USA | The experience of panic attack was determined through self-report questionnaire and not structured interview or other diagnosis. | PD definition ineligible |
| Rapsey ^37^ | Multi- country | The COPD variable was determined by self-report. | COPD definition ineligible |
| Spitzer ^38^ | Germany | The study reported odds ratios between panic disorder and COPD and no prevalence rates were reported. | Prevalence not reported |
| Wang ^39^ | USA | The sample overlapped the included study by Baker. | Overlapping sample |
| Yohannes ^40^ | UK | The study used the Geriatric Mental State Schedule^41^ for probable anxiety but did not report PD. | PD definition ineligible |

*COPD, chronic obstructive pulmonary disease; ICD-10, International Classification of Diseases 10 revision; PD, panic disorder; PRIME-MD, Primary Care Evaluation of Mental Disorders; SCL-90; Symptom Checklist-90*

**Supplement 5. Study characteristics pertaining to psychiatric classification and ratings**

| **Study ID** | **Psychiatric interview** | **Psychiatric classification** | **PD ± agoraphobia** | **Current PD** | **Lifetime PD** | **Rater blinding** | **PD in COPD qualifier** | **Rater qual.** |
| --- | --- | --- | --- | --- | --- | --- | --- | --- |
| Aghanwa 2001 ^5^ | PSE | ICD-10 | UC | UC | UC | No | NR | UC |
| Aydin 2001 ^14^ | CIDI | DSM-IV | NR | NR | NR | No | NR | Physician |
| Baker ^42^ | MINI 7 | DSM-5 | Separate | Yes | Yes | No | NR | Mixed |
| Chandel 2023 ^15a^ | MINI 5 | DSM-IV | UC | Yes | Yes | No | NR | Psychiatrist |
| Chaudhary 2016 ^43^ | MINI 6 | DSM-IV | UC | Yes | No | No | NR | UC |
| Dar 2019 ^44^ | MINI | DSM-IV | No | Yes | No | No | NR | Psychiatrist |
| Dowson 2010 ^45^ | MINI | DSM-IV | No | Yes | No | No | Excluded | UC |
| Karajgi 1990 ^46^ | SCID | DSM-III-R | Yes | Yes | No | No | Excluded | Physician |
| Kuhl 2008 ^47^ | UC | ICD-10 | And/ or | Yes | No | Yes | NR (blind) | Psychologist |
| Kunik 2009 ^48^ | SCID | DSM-IV | Yes | Yes | No | No | NR | UC |
| Laurin 2007 ^49^ | ADIS | DSM-IV | No | Yes | No | UC | NR | Psychologist |
| Livermore 2008 ^50^ | ADIS | DSM-IV | UC | Yes | No | No | NR | UC |
| Livermore 2012 ^51^ | ADIS | DSM-IV | No | Yes | No | No | NR | Psychologist |
| Mehta 2014 ^52^ | NR | DSM-5 | Separate | Yes | No | No | NR | Psychiatrist |
| Pascal 2017 ^53b^ | UC | ICD-10 | No | PA | No | UC | NR | UC |
| Pothirat 2015 ^54^ | MINI 5 | DSM-IV | NR | Yes | No | UC | NR | UC |
| Sharma ^55^ | MINI | DSM-5 | NR | Yes | No | No | NR | Psychiatrist |
| Singh ^56^ | MINI | DSM-5 | NR | Yes | No | UC | NR | UC |
| Sood 2024 ^57^ | MINI | ICD-10 | UC | Yes | No | UC | NR | UC |
| Vogele 2008 ^58^ | F-DIPS | DSM-IV | Yes | Yes | Yes | No | Excluded | Psychologist |
| Yellowlees ^59^ | NR | DSM-III | UC | Yes | No | No | NR | Psychiatrist |

*ADIS – IV, Anxiety Disorders Interview Schedule for DSM-IV; CIDI, Composite International Diagnostic Interview; DSM IV, Diagnostic and Statistical Manual of Mental Disorders, 4^th^ Edition; F-DIPS, Diagnostisches Interview für Psychische Störungen-Forschungsversion (Diagnostic Interview for Psychological Disorders Research Version); ICD -10, International Classification of Diseases, 10th Revision; MINI, MINI International Neuropsychiatric Interview; NR, not reported; PA, panic attacks; PD, panic disorder; SCID, Structured Clinical Interview for DSM; UC, unclear;*

1. *The methods stated that current and lifetime disorders were assessed ^15^ though Tables 1 to 5 do not differentiate between current and lifetime disorders, data are presumed to reflect current panic disorder.*
2. *Pascal 2017 reported panic attacks and not panic disorder ^53^.*

**Supplement 6. Psychiatric prevalence data for other conditions and medications**

| **Study ID** | **Major depression prevalence in COPD** | **Dysthymia prevalence in COPD** | | **Generalized Anxiety Disorder prevalence in COPD** | | **Prevalence of**  **antidepressant use in COPD** | | | **Prevalence of anxiolytic use in COPD** | | **Prevalence of any psychotropic medication use in COPD** |
| --- | --- | --- | --- | --- | --- | --- | --- | --- | --- | --- | --- |
| Aghanwa 2001 ^5^ | - | 16.7 | | - | | - | | | - | | - |
| Aydin 2001 ^14^ | 39.5 | - | | 15.8 | | - | | | - | | - |
| Baker ^42^ | - | - | | 1.8 | | - | | | 9.0 | | 30.0 |
| Chaudhary ^43^ | 2.70 | 5.41 | | 4.40 | |  | | |  | |  |
| Chandel 2023 ^15^ | 14.0 | - | | 20.0 | | 0 | | | 6.0 | | - |
| Dar 2019 ^44^ | 28.0 | 4.0 | | 2.0 | | - | | | - | | - |
| Dowson 2010 ^45^ | - | - | | - | | - | | | - | | - |
| Karajgi 1990 ^46^ | 6.0 | 8.0 | | - | | - | | | - | | - |
| Kuhl 2008 ^47^ | 11.9 | 2.1 | | 6.0 | | - | | | - | | - |
| Kunik 2009 ^48^ | 23.0 | 14.7 | | 19.1 | | 20.0 | | | 20.0 | | - |
| Laurin 2007 ^49^ | 12.9 | 1.7 | | 18.1 | | - | | | - | | - |
| Livermore 2008 ^50^ | - |  | | - | | - | | | 5.8 | |  |
| Livermore 2012 ^51^ | - | - | | - | | - | | | - | | - |
| Mehta 2014 ^52^ | 32.2 | - | | 1.7 | | - | | | - | | - |
| Pascal 2017 ^53^ | - | - | | - | | - | | | - | | - |
| Pothirat 2015 ^54^ | 12.2 | - | | 17.1 | | - | | | - | | - |
| Sharma ^55^ | 31.4 | 2.1 | | 23.6 | | - | | | - | | - |
| Singh ^56^ | 23.8 | | 2.5 | | 4.9 | | - | - | | - | |
| Sood 2024 ^57^ | - | | 26.0 | | 2.0 | | - | - | | - | |
| Vogele 2008 ^58^ | - | | 5.0 | | - | | - | - | | - | |

*COPD, chronic obstructive pulmonary disease*

**Supplement 7. Adjudication of case-control studies according to JBI checklist (item number in parentheses)**

| **Study ID** | **Group comparable (1)** | **Case control matching (2)** | **Criteria for case control, exposed unexposed (3)** | **Exposure valid, reliable (4, COPD)** | **Exposure measured the same (5, COPD)** | **Confounding factors identified (6, NA)** | **Confounding factor strategies (7, NA)** | **Outcome measure valid, reliable (8, PD)** | **F/Up sufficient (9, NA)** | **Analyses appropriate (10)** |
| --- | --- | --- | --- | --- | --- | --- | --- | --- | --- | --- |
| Aghanwa 2001 ^5^ | Yes | Yes | Yes | Yes | Yes | NA | NA | UC | NA | UC |
| Aydin 2001 ^14^ | No | No | Yes | UC | UC | NA | NA | Yes | NA | UC |
| Chaudhary ^43^ | UC | Yes | Yes | Yes | Yes | NA | NA | Yes | NA | Yes |
| Dar 2019 ^44^ | No | Yes | Yes | UC | Yes | NA | NA | Yes | NA | Yes |
| Kuhl 2008 ^47^ | UC | No | Yes | Yes | Yes | NA | NA | Yes | NA | Yes |
| Livermore 2008 ^50^ | UC | Yes | Yes | Yes | Yes | NA | NA | Yes | NA | Yes |
| Singh ^56^ | Yes | Yes | Yes | Yes | Yes | NA | NA | Yes | NA | Yes |
| Vogele 2008 ^58^ | Yes | No | Yes | Yes | Yes | NA | NA | Yes | NA | Yes |

*COPD, chronic obstructive pulmonary disease; F/Up, follow-up; JBI, Joanna Briggs Institute; NA, not applicable, PD, panic disorder; UC, unclear;*

***JBI case-control checklist items ^10^;***

*1. Were the groups comparable other than presence of disease in cases or absence of disease in controls?*

*2. Were cases and controls matched appropriately?*

*3. Were the same criteria used for identification of cases and controls?*

*4. Was exposure measured in a standard, valid and reliable way?*

*5. Was exposure measured in the same way for cases and controls?*

*6. Were confounding factors identified?*

*7. Were strategies to deal with confounding factors stated?*

*8. Were outcomes assessed in a standard, valid and reliable way for cases and controls?*

*9. Was the exposure period of interest long enough to be meaningful?*

*10. Was appropriate statistical analysis used?*

**Supplement 8 Adjudication of study quality in cross-sectional analytical studies according to JBI checklist (item number in parentheses)**

| **Study ID** | **Inclusion defined (1)** | **Population and setting (2)** | **Exposure measurement (3, COPD)** | **Measurement of condition (4, COPD)** | **Confounding identified (5, NA)** | **Confounding strategies (6, NA)** | **Outcome reliable (7, PD)** | **Appropriate analysis (8)** |
| --- | --- | --- | --- | --- | --- | --- | --- | --- |
| Baker ^42^ | Yes | Yes | Yes | Yes | NA | NA | Yes | Yes |
| Chandel 2023 ^15^ | No | No | No | No | NA | NA | Yes | UC |
| Dowson 2010 ^45^ | Yes | Yes | Yes | Yes | NA | NA | Yes | Yes |
| Karajgi 1990 ^46^ | Yes | Yes | Yes | Yes | NA | NA | Yes | Yes |
| Kunik 2009 ^48^ | Yes | Yes | Yes | Yes | NA | NA | Yes | Yes |
| Laurin 2007 ^49^ | Yes | Yes | Yes | Yes | NA | NA | Yes | Yes |
| Livermore 2012 ^51^ | Yes | Yes | Yes | Yes | NA | NA | Yes | Yes |
| Mehta 2014 ^52^ | Yes | Yes | Yes | Yes | NA | NA | Yes | Yes |
| Pascal 2017 ^53^ | Yes | Yes | Yes | Yes | NA | NA | UC | Yes |
| Pothirat 2015 ^54a^ | Yes | Yes | Yes | Yes | NA | NA | Yes | Yes |
| Sharma ^55^ | No | Yes | Yes | Yes | NA | NA | Yes | Yes |
| Sood 2024 ^57^ | Yes | Yes | Yes | Yes | NA | NA | Yes | Yes |
| Yellowlees ^59^ | Yes | Yes | Yes | Yes | NA | NA | Yes | Yes |

1. *This study was cross-sectional and not designed as case-control however we extracted data from non COPD control groups*

*COPD, chronic obstructive pulmonary disease; JBI, Joanna Briggs Institute; NA, not applicable, PD, panic disorder; UC, unclear;*

***JBI analytical cross-sectional checklist items;***

*1. Were the criteria for inclusion in the sample clearly defined?*

*2. Were the study subjects and the setting described in detail?*

*3. Was the exposure measured in a valid and reliable way?*

*4. Were objective, standard criteria used for measurement of the condition?*

*5. Were confounding factors identified?*

*6. Were strategies to deal with confounding factors stated?*

## *
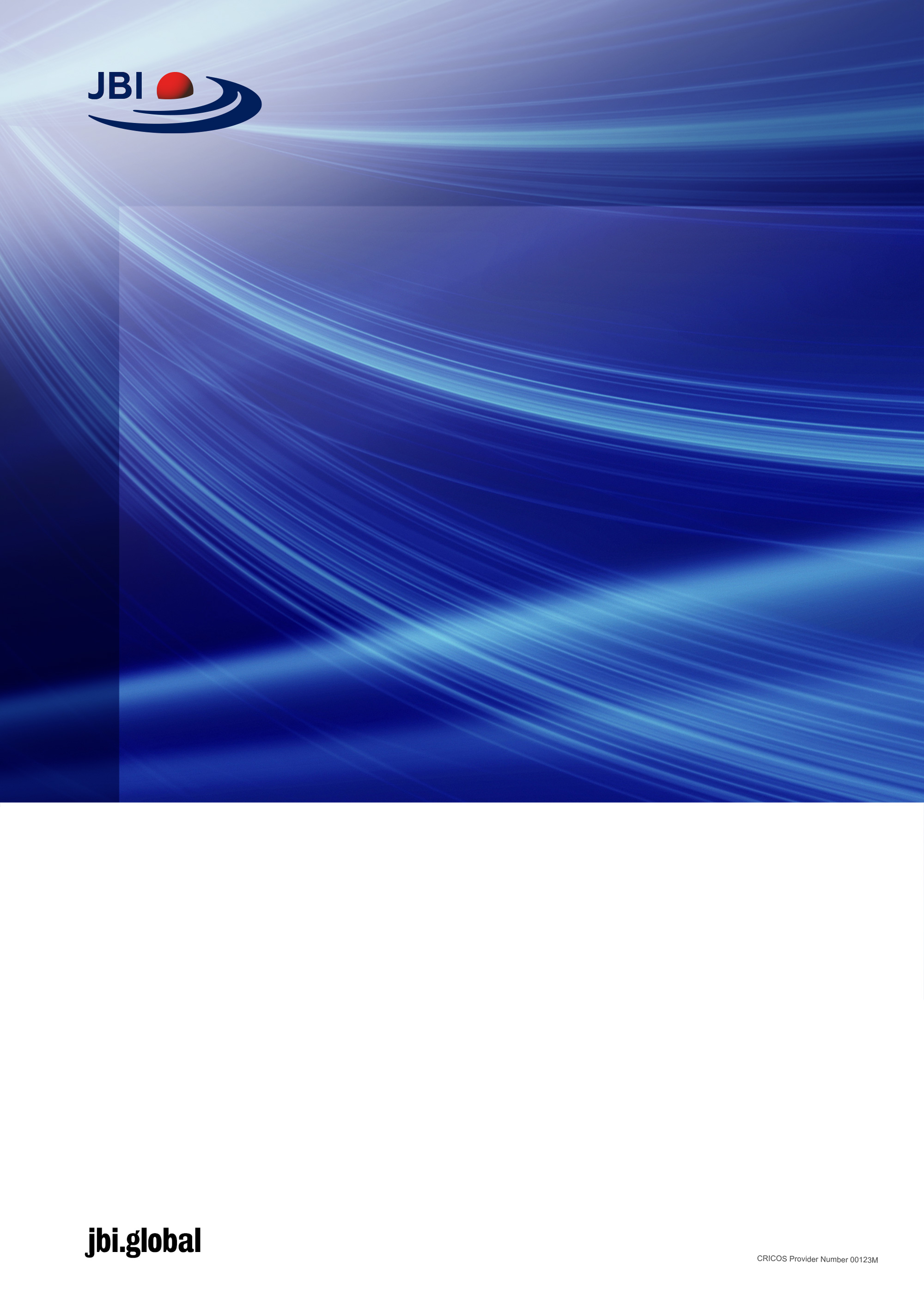
7. Were the outcomes measured in a valid and reliable way?*

## *8. Was appropriate statistical analysis used?*

**Supplement 9. Bayesian prediction intervals for panic disorder prevalence in COPD**

A. With outliers

B. Outliers excluded

Outlier studies ^45 49 51 53 58^.

**Supplement 10. Funnel plots to ascertain publication bias**

*A. With outliers*

*B. Outliers excluded*

Non-shaded circles represent published effect sizes

Shaded circles represent imputed effect sizes

Plot shows the logit event rate (x axis) plotted by the standard error (y axis)

Outlier studies ^45 49 51 53 58^.

**Supplement 11.** **Tests of publication bias for the prevalence of panic disorder in COPD**

|  | **With Outliers k=21** | **Outliers Excluded k=16** |
| --- | --- | --- |
| Begg-Mazumdar^a^ | τ = -0.26, z = 1.66, p = 0.097 | τ = -0.29, z = 1.58, p = 0.12 |
| Egger | Intercept -5.10 (95% CI -8.09 to -2.10)  t(19) = 3.56, p = 0.002 | Intercept -3.05 (95% CI -5.38 to -0.71)  t(14) = 2.80, p = 0.014 |
| Duval and Tweedie – adjusted^b^ | Q = 230.08, n = 4 adjusted | Q = 80.65, n = 5 adjusted |
| Observed prevalence | 12.5 (95% CI 8.2 – 18.8) | 8.1 (95% CI 5.7 – 11.6) |
| Adjusted prevalence (trimmed) ^b^ | 16.1 (95% CI 10.8 – 22.3 | 10.6 (95% CI 7.4 – 15.0) |

^a^ Kendall’s tau with continuity correction and p (two-tailed). ^b^ Random effects, imputed right of mean

CI, confidence interval; COPD, chronic obstructive pulmonary disease;

Outlier studies ^45 49 51 53 58^.

**Supplement 12.** **Forest plots showing the change in panic disorder prevalence when one study was removed from analysis**

*A. With outliers*

*B. Outliers excluded*

Outlier studies ^45 49 51 53 58^.

**Supplement 13. Meta-regression and moderator analysis for continuous variables**

*COPD, chronic obstructive pulmonary disease; FEV, forced expiratory volume; FVC, forced vital capacity; GAD, generalized anxiety disorder; MDD, major depressive disorder;*

Outlier studies ^45 49 51 53 58^.

| Model | All studies | | | | | | | |  | Outliers excluded^a^ | | | | | | | |
| --- | --- | --- | --- | --- | --- | --- | --- | --- | --- | --- | --- | --- | --- | --- | --- | --- | --- |
|  | Covariate in model | | | Goodness of fit | | | | |  | Covariate in model | | | Model Goodness of fit | | | | |
|  | *β* | *95% CI* | *P* | *T^2^* | *I*^2^ | *Q* | *df* | *P* |  | *β* | *95% CI* | *P* | *T^2^* | *I*^2^ | *Q* | *df* | *P* |
| Bivariate |  |  |  |  |  |  |  |  |  |  |  |  |  |  |  |  |  |
| Mean age, yrs | .02 | -.07 to .10 | .70 | 4.44 | 89 | 168.1 | 18 | <.001 |  | .002 | -.064 to .07 | 0.96 | .532 | 77 | 55.2 | 13 | <.001 |
| Male sex % | -.02 | -.05 to .01 | .26 | - | - | - | - | - |  | -.003 | -.031 to .03 | 0.83 | - | - | - | - | - |
| Univariate |  |  |  |  |  |  |  |  |  |  |  |  |  |  |  |  |  |
| FEV₁/ FVC | 8.54 | -18.1 to 35.1 | .53 | 1.57 | 93 | 89.8 | 6 | <.001 |  | 14.1 | -2.80 to 30.9 | .10 | .21 | 55 | 6.6 | 3 | .08 |
| FEV_1_ pred | .04 | -.03 to .11 | .31 | 1.17 | 92 | 114.7 | 9 | <.001 |  | .03 | -.07 to .13 | .54 | .40 | 72 | 17.8 | 5 | <.01 |
| Smoking, % | .00 | -.03 to .04 | .84 | 1.37 | 92 | 139.2 | 11 | <.001 |  | -.01 | -.04 to .03 | .72 | .61 | 81 | 36.6 | 7 | <.001 |
| COPD yrs | .06 | -.20 to .31 | .65 | 1.80 | 92 | 86.1 | 7 | <.001 |  | .07 | -.43 to .57 | .79 | 1.20 | 89 | 26.7 | 3 | <.001 |
| MDD, % | -.02 | -.07 to .03 | .45 | .54 | 86 | 79.4 | 11 | <.001 |  | -.007 | -.043 to .03 | .69 | .28 | 70 | 33.6 | 10 | <.001 |
| GAD, % | .04 | -.02 to .10 | .22 | .71 | 86 | 87.1 | 12 | <.001 |  | -.005 | -.062 to .05 | .86 | .36 | 73 | 30.0 | 8 | <.001 |

**Supplement 14. Sub-group moderator analyses for categorical variables**

|  | **With Outliers k=21** | | | | | **Outliers Excluded k=16** | | | | |
| --- | --- | --- | --- | --- | --- | --- | --- | --- | --- | --- |
| **Subgroup Analysis** | **k** | **Prevalence** | **95% CI** | **I^2^** | **P for difference** | **k** | **Prevalence** | **95% CI** | **I^2^** | **P for difference** |
| Region |  |  |  |  | <0.001 |  |  |  |  | <0.001 |
| Africa | 1 | 3.3 | 0.5 – 20.2 | - |  | 1 | 3.3 | 0.5 – 20.2 | - |  |
| Asia | 9 | 8.0 | 5.0 – 12.6 | 69 |  | 9 | 8.0 | 5.0 – 12.6 | 69 |  |
| Europe | 3 | 25.7 | 7.1 – 61.0 | 94 |  | 1 | 7.7 | 4.3 – 13.4 | 0 |  |
| North America | 4 | 9.1 | 2.0 – 32.8 | 96 |  | 3 | 5.0 | 2.8 – 8.7 | 44 |  |
| Oceania | 4 | 29.8 | 20.5 – 41.1 | 67 |  | 2 | 20.8 | 14.0 – 29.8 | 0 |  |
| Study design |  |  |  |  | <0.001 |  |  |  |  | 0.89 |
| Case-control | 7 | 9.8 | 4.9 – 18.5 | 76 |  | 6 | 8.0 | 4.8 – 12.9 | 46 |  |
| Cross-sectional | 13 | 12.8 | 7.4 – 21.3 | 92 |  | 10 | 8.4 | 5.1 – 13.5 | 80 |  |
| Cohort | 1 | 36.8 | 26.8 – 48.2 | - |  | - | - | - | - |  |
| Sampling |  |  |  |  | 0.066 |  |  |  |  | 0.64 |
| General community | 3 | 5.6 | 2.9 – 10.5 | 66 |  | 3 | 5.6 | 2.9 – 10.5 | 66 |  |
| In-patients | 5 | 18.9 | 8.5 – 36.8 | 80 |  | 3 | 6.2 | 0.8 – 34.1 | 82 |  |
| Mix in- out-patients | 3 | 10.0 | 4.0 – 22.9 | 78 |  | 3 | 10.0 | 4.0 – 22.9 | 78 |  |
| Outpatients | 10 | 14.8 | 8.1 – 25.5 | 91 |  | 7 | 8.8 | 5.7 – 13.2 | 55 |  |
| Subjective COPD assessments |  |  |  |  | 0.67 |  |  |  |  | 0.84 |
| No | 15 | 11.8 | 7.2 - 18.8 | 87 |  | 12 | 8.3 | 5.3 - 12.7 | 74 |  |
| Yes | 6 | 14.6 | 5.9 - 31.6 | 94 |  | 4 | 7.6 | 3.8 - 14.6 | 75 |  |
| Psychiatric Interview |  |  |  |  | <0.001 |  |  |  |  | 0.036 |
| ADIS | 3 | 32.1 | 20.7 - 46.1 | 76 |  | 1 | 17.3 | 9.3 – 30.0 | - |  |
| CIDI | 1 | 2.6 | 0.4 - 16.5 | - |  | 1 | 2.6 | 0.4 – 16.5 | - |  |
| F-DIPS | 1 | 40.0 | 21.4 - 62.0 | - |  | - | - | - | - |  |
| MINI | 9 | 7.9 | 3.9 - 15.3 | 89 |  | 8 | 6.4 | 3.6 – 11.2 | 79 |  |
| PSE | 1 | 3.3 | 0.5 - 20.2 | - |  | 1 | 3.3 | 0.5 – 20.2 | - |  |
| SCID | 2 | 6.3 | 3.9 - 10.1 | 0 |  | 2 | 6.3 | 3.9 – 10.1 | 0 |  |
| Unclear | 4 | 19.9 | 8.4 - 40.1 | 90 |  | 3 | 14.3 | 7.1 – 26.8 | 77 |  |
| Classification |  |  |  |  | 0.76 |  |  |  |  | 0.45 |
| DSM-III | 2 | 15.0 | 4.8 - 38.3 | 77 |  | 2 | 15.0 | 4.8 – 38.3 | 77 |  |
| DSM-IV | 11 | 14.7 | 8.1 – 25.3 | 90 |  | 7 | 7.6 | 4.8 – 11.8 | 51 |  |
| DSM-5 | 4 | 9.2 | 4.0 – 19.7 | 87 |  | 4 | 9.2 | 4.0 – 19.7 | 87 |  |
| ICD-10 | 4 | 9.1 | 1.9 – 34.6 | 94 |  | 3 | 5.4 | 2.8 – 10.3 | 24 |  |
| Agoraphobia reported |  |  |  |  | 0.33 |  |  |  |  | 0.50 |
| No | 15 | 14.4 | 9.0 – 22.0 | 89 |  | 11 | 8.8 | 5.6 – 13.7 | 72 |  |
| Yes | 6 | 9.6 | 4.7 – 18.6 | 84 |  | 5 | 7.0 | 4.1 – 11.6 | 67 |  |
| Lifetime panic reported |  |  |  |  | 0.064 |  |  |  |  | 0.002 |
| No | 16 | 14.8 | 9.7 - 22.1 | 90 |  | 12 | 10.0 | 7.1 – 13.9 | 71 |  |
| Unclear | 2 | 3.0 | 0.7 – 11.1 | 0 |  | 2 | 3.0 | 0.7 – 11.1 | 0 |  |
| Yes | 3 | 7.2 | 0.7 – 46.4 | 93 |  | 2 | 2.6 | 1.3 – 5.4 | 0 |  |
| Panic modified/ blinded |  |  |  |  | 0.33 |  |  |  |  | 0.99 |
| Modified | 3 | 24.0 | 7.5 – 55.0 | 93 |  | 1 | 8.0 | 3.0 – 19.5 | - |  |
| Blinded | 1 | 8.0 | 0.3 – 19.5 | - |  | 1 | 7.7 | 4.3 – 13.4 | - |  |
| None | 17 | 11.2 | 6.8 – 17.8 | 90 |  | 14 | 8.1 | 5.3 – 12.1 | 76 |  |
| Qualifications |  |  |  |  | <0.001 |  |  |  |  | <0.01 |
| Mixed | 1 | 2.7 | 1.2 – 6.0 | - |  | 1 | 2.7 | 1.2 – 6.0 | - |  |
| Physician | 2 | 6.3 | 2.5 – 14.8 | 5 |  | 2 | 6.3 | 2.5 – 14.8 | 5 |  |
| Psychiatrist | 5 | 14.4 | 8.9 – 22.4 | 65 |  | 5 | 14.4 | 8.9 – 22.4 | 65 |  |
| Psychologist | 4 | 28.0 | 13.1 – 50.0 | 91 |  | 1 | 7.7 | 4.3 – 13.4 | - |  |
| Unclear | 9 | 10.8 | 5.1 – 21.6 | 91 |  | 7 | 7.1 | 4.6 – 11.0 | 53 |  |

ADIS, Anxiety Disorders Interview Schedule for DSM-IV; CI, confidence interval; CIDI, Composite International Diagnostic Interview; COPD, chronic obstructive pulmonary disease; DSM, Diagnostic and Statistical Manual of Mental Disorders; F-DIPS, Diagnostisches Interview für Psychische Störungen-Forschungsversion (Diagnostic Interview for Psychological Disorders Research Version); ICD -10, International Classification of Diseases, 10th Revision; MINI, MINI International Neuropsychiatric Interview;

PSE, Present State Examination; SCID, Structured Clinical Interview for DSM;

Outlier studies ^45 49 51 53 58^.

**Supplement 15. References**

1. Page MJ, McKenzie JE, Bossuyt PM, et al. The PRISMA 2020 statement: an updated guideline for reporting systematic reviews. *BMJ* 2021;372:n71. doi: 10.1136/bmj.n71

2. Global Initiative for Chronic Obstructive Lung Disease (GOLD). Global Strategy for Prevention, Diagnosis and Management of COPD: 2024 Report, 2024.

3. Page MJ, McKenzie JE, Bossuyt PM, et al. The PRISMA 2020 statement: an updated guideline for reporting systematic reviews. *Systematic Reviews* 2021;10(1):89. doi: 10.1186/s13643-021-01626-4

4. Qiu CJ, Wu S. Depression and anxiety disorders in chronic obstructive pulmonary disease patients: Prevalence, disease impact, treatment. *World J Psychiatry* 2024;14(12):1797-803. doi: 10.5498/wjp.v14.i12.1797 [published Online First: 20241219]

5. Willgoss TG, Yohannes AM. Anxiety disorders in patients with COPD: a systematic review. *Respiratory Care* 2013;58(5):858-66. doi: 10.4187/respcare.01862

6. Barrera TL, Grubbs KM, Kunik ME, et al. A review of cognitive behavioral therapy for panic disorder in patients with chronic obstructive pulmonary disease: the rationale for interoceptive exposure. *J Clin Psychol Med Settings* 2014;21(2):144-54. doi: 10.1007/s10880-014-9393-4

7. Livermore N, Sharpe L, McKenzie D. Panic attacks and panic disorder in chronic obstructive pulmonary disease: a cognitive behavioral perspective. *Respiratory medicine* 2010;104(9):1246-53. doi: 10.1016/j.rmed.2010.04.011 [published Online First: 20100508]

8. Bramer WM, Giustini D, de Jonge GB, et al. De-duplication of database search results for systematic reviews in EndNote. *J Med Libr Assoc* 2016;104(3):240-3. doi: 10.3163/1536-5050.104.3.014

9. Clark J, Glasziou P, Del Mar C, et al. A full systematic review was completed in 2 weeks using automation tools: a case study. *J Clin Epidemiol* 2020;121:81-90. doi: 10.1016/j.jclinepi.2020.01.008 [published Online First: 20200128]

10. Joanna Briggs Institute. JBI Manual for Evidence Synthesis In: Aromataris E, Munn Z, eds., 2020.

11. Borenstein M, Hedges L, Higgins J, et al. Comprehensive Meta-Analysis Version 4. 2014.

12. Tully PJ, Cosh SM, Baumeister H. The anxious heart in whose mind? A systematic review and meta-regression of factors associated with anxiety disorder diagnosis, treatment and morbidity risk in coronary heart disease. *Journal of Psychosomatic Research* 2014;77(6):439-48. doi: 10.1016/j.jpsychores.2014.10.001

13. Aghanwa HS, Erhabor GE. Specific psychiatric morbidity among patients with chronic obstructive pulmonary disease in a Nigerian general hospital. *Journal of Psychosomatic Research* 2001;50(4):179-83. doi: <https://doi.org/10.1016/S0022-3999(00)00206-3>

14. Aydin IO, Uluşahin A. Depression, anxiety comorbidity, and disability in tuberculosis and chronic obstructive pulmonary disease patients: applicability of GHQ-12. *General hospital psychiatry* 2001;23(2):77-83. doi: <https://doi.org/10.1016/S0163-8343(01)00116-5>

15. Chandel RK, Menia A, Mitla V. Prevalence of psychiatric disorders in patients with COPD in a tertiary healthcare setting. *Journal of Cardiovascular Disease Research* 2023;14(1):3623-2629.

16. Egger M, Davey SG, Schneider M, et al. Bias in meta-analysis detected by a simple, graphical test *BMJ* 1997;315 (315(7109)):629 –34. doi: DOI: 10.1136/bmj.315.7109.629

17. Begg CB, Mazumdar M. Operating characteristics of a rank correlation test for publication bias. *Biometrics* 1994;50 1088 –101.

18. Duval S, Tweedie R. Trim and fill: A simple funnel-plot-based method of testing and adjusting for publication bias in meta-analysis. *Biometrics* 2000;56(2):455-63. [published Online First: 2000/07/06]

19. Altman D, Ashby D, Birks J, et al. Chapter 10: Analysing data and undertaking meta-analyses. In: Jonathan J Deeks JPH, Douglas G Altman, Joanne E McKenzie and Areti Angeliki Veroniki; on behalf of the Cochrane Statistical Methods Group, ed. Cochrane Handbook for Systematic Reviews of Interventions, 2024.

20. Higgins JPT, Thomas J, Chandler J, et al. Cochrane Handbook for Systematic Reviews of Interventions version 6.3 (updated February 2022) In: Cochrane, ed. Available from <www.training.cochrane.org/handbook>., 2022.

21. Chan HN, Yap HL, Kanagasuntheram N, et al. Prevalence of depression and anxiety in hospitalized chronic obstructive pulmonary disease patients and their quality of life: a pilot study. *Asia-Pacific Psychiatry* 2009;1(3):130-37. doi: <https://doi.org/10.1111/j.1758-5872.2009.00040.x>

22. Chetty U, McLean G, Morrison D, et al. Chronic obstructive pulmonary disease and comorbidities: a large cross-sectional study in primary care. *Br J Gen Pract* 2017;67(658):e321-e28. doi: 10.3399/bjgp17X690605

23. Dua R, Das A, Kumar A, et al. Association of comorbid anxiety and depression with chronic obstructive pulmonary disease. *Lung India* 2018;35:31-36.

24. El-Gabalawy R, Mackenzie CS, Shooshtari S, et al. Comorbid physical health conditions and anxiety disorders: a population-based exploration of prevalence and health outcomes among older adults. *General hospital psychiatry* 2011;33(6):556-64. doi: 10.1016/j.genhosppsych.2011.07.005 [published Online First: 20110909]

25. Garg P, Kumar S, Kumar A, et al. Study of psychiatric co-morbidity and quality of life in individuals suffering from chronic lung disease. *Journal of Cardiovascular Disease Research* 2023;14(6):2650-.

26. Giardino ND, Curtis JL, Abelson JL, et al. The impact of panic disorder on interoception and dyspnea reports in chronic obstructive pulmonary disease. *Biological psychology* 2010;84(1):142-6. doi: 10.1016/j.biopsycho.2010.02.007 [published Online First: 20100220]

27. Holas P, Michałowski J, Gawęda Ł, et al. Agoraphobic avoidance predicts emotional distress and increased physical concerns in chronic obstructive pulmonary disease. *Respiratory medicine* 2017;128:7-12. doi: 10.1016/j.rmed.2017.04.011 [published Online First: 20170421]

28. Howard C, Hallas CN, Wray J, et al. The relationship between illness perceptions and panic in chronic obstructive pulmonary disease. *Behaviour Research and Therapy* 2009;47(1):71-76. doi: <https://doi.org/10.1016/j.brat.2008.10.004>

29. Hsieh MH, Lee CT, Tsai PJ, et al. Chronic obstructive pulmonary disease and anxiety disorders: a nationwide population-based study in Taiwan. *General hospital psychiatry* 2016;42:1-6. doi: 10.1016/j.genhosppsych.2016.05.005 [published Online First: 20160531]

30. Livermore N, Sharpe L, McKenzie D. Prevention of panic attacks and panic disorder in COPD. *European Respiratory Journal* 2010;35(3):557-63. doi: 10.1183/09031936.00060309

31. Moore MC, Zebb BJ. The catastrophic misinterpretation of physiological distress. *Behav Res Ther* 1999;37(11):1105-18. doi: 10.1016/s0005-7967(98)00197-1

32. Moretta P, Cavallo ND, Candia C, et al. Psychiatric Disorders in Patients with Chronic Obstructive Pulmonary Disease: Clinical Significance and Treatment Strategies. *Journal of Clinical Medicine* 2024;13(21):6418.

33. Ohayon MM. Chronic Obstructive Pulmonary Disease and its association with sleep and mental disorders in the general population. *J Psychiatr Res* 2014;54:79-84. doi: 10.1016/j.jpsychires.2014.02.023 [published Online First: 20140314]

34. Perna G, Bertani A, Diaferia G, et al. Prevalence of respiratory diseases in patients with panic and obsessive compulsive disorders. *Anxiety* 1994;1(2):100-1. doi: 10.1002/anxi.3070010210

35. Pollack MH, Kradin R, Otto MW, et al. Prevalence of panic in patients referred for pulmonary function testing at a major medical center. *Am J Psychiatry* 1996;153(1):110-3. doi: 10.1176/ajp.153.1.110

36. Porzelius J, Vest M, Nochomovitz M. Respiratory function, cognitions, and panic in chronic obstructive pulmonary patients. *Behav Res Ther* 1992;30(1):75-7. doi: 10.1016/0005-7967(92)90101-l

37. Rapsey CM, Lim CC, Al-Hamzawi A, et al. Associations between DSM-IV mental disorders and subsequent COPD diagnosis. *J Psychosom Res* 2015;79(5):333-9. doi: 10.1016/j.jpsychores.2015.08.005 [published Online First: 20150902]

38. Spitzer C, Gläser S, Grabe HJ, et al. Mental health problems, obstructive lung disease and lung function: Findings from the general population. *Journal of Psychosomatic Research* 2011;71(3):174-79. doi: <https://doi.org/10.1016/j.jpsychores.2011.03.005>

39. Wang JG, Bose S, Holbrook JT, et al. Clinical Characteristics of Patients With COPD and Comorbid Depression and Anxiety: Data From a National Multicenter Cohort Study. *Chronic Obstr Pulm Dis* 2025;12(1):33-42. doi: 10.15326/jcopdf.2024.0534

40. Yohannes AM, Baldwin RC, Connolly MJ. Depression and anxiety in elderly outpatients with chronic obstructive pulmonary disease: prevalence, and validation of the BASDEC screening questionnaire. *Int J Geriatr Psychiatry* 2000;15(12):1090-6. doi: 10.1002/1099-1166(200012)15:12<1090::aid-gps249>3.0.co;2-l

41. Copeland JR, Kelleher MJ, Kellett JM, et al. A semi-structured clinical interview for the assessment of diagnosis and mental state in the elderly: the Geriatric Mental State Schedule. I. Development and reliability. *Psychol Med* 1976;6(3):439-49. doi: 10.1017/s0033291700015889

42. Baker AM, Holbrook JT, Yohannes AM, et al. Test Performance Characteristics of the AIR, GAD-7, and HADS-Anxiety Screening Questionnaires for Anxiety in Chronic Obstructive Pulmonary Disease. *Ann Am Thorac Soc* 2018;15(8):926-34. doi: 10.1513/AnnalsATS.201708-631OC

43. Chaudhary SC, Nanda S, Tripathi A, et al. Prevalence of psychiatric comorbidities in chronic obstructive pulmonary disease patients. *Lung India* 2016;33(2):174-8. doi: 10.4103/0970-2113.177441

44. Dar SA, Bhat BA, Shah NN, et al. The Pattern of Psychiatric Morbidity in Chronic Obstructive Pulmonary Disease: A Cross-Sectional, Case-Control Study from a Tertiary Care Hospital in Kashmir, North India. *J Neurosci Rural Pract* 2019;10(1):65-70. doi: 10.4103/jnrp.jnrp_244_18

45. Dowson CA, Kuijer RG, Town IG, et al. Impact of panic disorder upon self-management educational goals in chronic obstructive pulmonary disease? *Chron Respir Dis* 2010;7(2):83-90. doi: 10.1177/1479972310365363 [published Online First: 20100318]

46. Karajgi B, Rifkin A, Doddi S, et al. The prevalence of anxiety disorders in patients with chronic obstructive pulmonary disease. *Am J Psychiatry* 1990;147(2):200-1. doi: 10.1176/ajp.147.2.200

47. Kühl K, Schürmann W, Rief W. Mental disorders and quality of life in COPD patients and their spouses. . *Int J Chron Obstruct Pulmon Dis* 2008;3(4):727-36. doi: <https://doi.org/10.2147/COPD.S3375>

48. Kunik ME, Roundy K, Veazey C, et al. Surprisingly high prevalence of anxiety and depression in chronic breathing disorders. *Chest* 2005;127(4):1205-11. doi: 10.1378/chest.127.4.1205

49. Laurin C, Lavoie KL, Bacon SL, et al. Sex Differences in the Prevalence of Psychiatric Disorders and Psychological Distress in Patients With COPD. *Chest* 2007;132(1):148-55. doi: <https://doi.org/10.1378/chest.07-0134>

50. Livermore N, Butler JE, Sharpe L, et al. Panic attacks and perception of inspiratory resistive loads in chronic obstructive pulmonary disease. *Am J Respir Crit Care Med* 2008;178(1):7-12. doi: 10.1164/rccm.200711-1700OC [published Online First: 20080424]

51. Livermore N, Sharpe L, McKenzie D. Catastrophic interpretations and anxiety sensitivity as predictors of panic-spectrum psychopathology in chronic obstructive pulmonary disease. *J Psychosom Res* 2012;72(5):388-92. doi: 10.1016/j.jpsychores.2012.02.001 [published Online First: 20120225]

52. Mehta JR, Ratnani IJ, Dave JD, et al. Association of psychiatric co-morbidities and quality of life with severity of chronic obstructive pulmonary disease. *East Asian Arch Psychiatry* 2014;24(4):148-55.

53. Pascal OI, Trofor AC, Lotrean LM, et al. Depression, anxiety and panic disorders in chronic obstructive pulmonary disease patients: correlations with tobacco use, disease severity and quality of life. *Tob Induc Dis* 2017;15:23. doi: 10.1186/s12971-017-0128-9 [published Online First: 20170407]

54. Pothirat C, Chaiwong W, Phetsuk N, et al. Major affective disorders in chronic obstructive pulmonary disease compared with other chronic respiratory diseases. *Int J Chron Obstruct Pulmon Dis* 2015;10:1583-90. doi: 10.2147/copd.S86742 [published Online First: 20150807]

55. Sharma S, Dixit S, Agrawal R, et al. Psychiatric Comorbidity and Quality of Life in Individuals with Chronic Obstructive Pulmonary Disease: A Cross-Sectional Study. *International Journal of Pharmaceutical and Clinical Research* 2024;16(9):471-76.

56. Singh P, Chaudhari B, Chaudhury S, et al. A study of psychiatric co-morbidities, anxiety, depression and quality of life in patients of chronic obstructive pulmonary disease. *Medical Journal of Dr DY Patil Vidyapeeth* 2024;17:24-33. doi: 10.4103/mjdrdypu.mjdrdypu_637_20

57. Sood R, Ahuja G, Sharma S, et al. Psychiatric comorbidities in patients with chronic obstructive pulmonary disease. *Int J Curr Pharm Res* 2024:38-51. doi: 10.22159/ijcpr.2024v16i2.4026

58. Vögele C, von Leupoldt A. Mental disorders in chronic obstructive pulmonary disease (COPD). *Respiratory medicine* 2008;102(5):764-73. doi: 10.1016/j.rmed.2007.12.006 [published Online First: 20080128]

59. Yellowlees PM, Alpers JH, Bowden JJ, et al. Psychiatric morbidity in patients with chronic airflow obstruction. *Med J Aust* 1987;146(6):305-7. doi: 10.5694/j.1326-5377.1987.tb120267.x
